# Heart Failure with Preserved Ejection Fraction Is a Still Big Unmet Need in Cardiology

**DOI:** 10.3390/jcm10112460

**Published:** 2021-06-01

**Authors:** Nobuyuki Ohte

**Affiliations:** Nagoya City University West Medical Center, 1-2-23 Wakamizu, Chikusa-ku, Nagoya 464-8547, Japan; ohte@med.nagoya-cu.ac.jp

Miñana and Núñez wrote an editorial to introduce the contents in a Special Issue of the Journal of Clinical Medicine focused on “Epidemiology, Diagnosis, Pathophysiology, Risk Stratification, and Therapy of Heart Failure with Preserved Ejection Fraction” [1]. Here, the author addresses his concerns in heart failure with preserved ejection fraction (HFpEF) as another editorial.

In patients with heart failure (HF), left ventricular (LV) diastolic dysfunction mainly causes HF symptoms such as dyspnea, irrespective of LV ejection fraction (EF). An elevated LV filling pressure due to LV diastolic dysfunction produces dyspnea at rest and on exercise [2]. Even when LV filling pressure is within the normal range at rest, exercise-induced hypertension may impair LV relaxation and provoke an increase in LV diastolic stiffness. Tachycardia and/or myocardial ischemia during exercise also bring an increased LV filling pressure through deteriorated left atrial conduit function and LV stiffening, respectively. In patients with HFpEF, abnormal LV relaxation has been emphasized as an important cause of dyspnea in patients with HFpEF [3].

The pathogenesis of HFpEF is multifactorial. It has been recognized that in addition to LV diastolic dysfunction caused by myocardial abnormality, a disorder in the LV-aortic coupling, atrial fibrillation, functional mitral regurgitation, and extracardiac comorbidities such as aging, female sex, hypertension, diabetes, chronic kidney disease, chronic obstructive lung disease, anemia, obesity, and frailty, may contribute to the HFpEF pathophysiology [4]; however, the relationships between LV diastolic dysfunction and these pathological conditions have not been adequately assessed in clinical settings. 

Interests regarding HFpEF maybe how to diagnose LV diastolic dysfunction noninvasively and how to treat HFpEF with evidence-based.

Noninvasive diagnosis of LV diastolic dysfunction in patients with preserved LVEF should be performed using the American Society of Echocardiography/European Association of Cardiovascular Imaging (ASE/EACVI) recommendations A and B as a standard manner [5]. The ASE/EACVI recommendation A is applied for screening LV diastolic dysfunction in patients with suspected HF symptoms and with normal LVEF. The ASE/EACVI recommendation B is effective for evaluating LV filling pressure mainly in patients with LV systolic dysfunction; however, it also works in patients with normal LVEF and with LV hypertrophy, prior myocardial infarction, and/or in those that fit the ASE/EACVI recommendation A positive. However, the utility of recommendation A is limited in sensitivity to identify LV diastolic dysfunction [6]. The utility of recommendation B was also limited in HFpEF patients compared with patients with HF and reduced LVEF [7]. Noninvasive diagnosis of LV diastolic dysfunction in patients with preserved LVEF is still challenging. The evaluation of left atrial strain may bring further progress for this purpose [8]. A diastolic stress test has high accuracy in diagnosing HFpEF [9]; however, it is time and human resource consuming, and is not suitable for all patients in whom HFpEF is suspected. 

In drug therapy for HFpEF, a couple of drugs have been noticed to have beneficial effects in the HFpEF patients with LVEF approximately <60% [10,11]. LVs with EF <60% may have different natures in LV contractile performance compared with that of EF ≥60%, even in patients who were sorted into HFpEF [12,13]. There may be mild myocardial damage in the LVs in patients with LVEF between 50 and 60%. Nowadays, HF with mid-range EF (HFmrEF) is defined as HF with LVEF between 40 and 50%. The upper LVEF value in HFmrEF might be reconsidered in the future.

## References

[1] Miñana G., Núñez J. (2021). Heart Failure with Preserved Ejection Fraction: An Urgent Need for Precision Medicine. J. Clin. Med..

[2] Kitzman D.W., Higginbotham M.B., Cobb F.R., Sheikh K.H., Sullivan M.J. (1991). Exercise intolerance in patients with heart failure and preserved left ventricular systolic function: Failure of the Frank-Starling mechanism. J. Am. Coll. Cardiol..

[3] Hatle L. (2007). How to diagnose diastolic heart failure a consensus statement. Eur. Heart J..

[4] Senni M., Paulus W.J., Gavazzi A., Fraser A.G., Díez J., Solomon S.D., Smiseth O.A., Guazzi M., Lam C.S.P., Maggioni A.P. (2014). New strategies for heart failure with preserved ejection fraction: The importance of targeted therapies for heart failure phenotypes. Eur. Heart J..

[5] Nagueh S.F., Smiseth O.A., Appleton C.P., Byrd B.F., Dokainish H., Edvardsen T., Flachskampf F.A., Gillebert T.C., Klein A.L., Lancellotti P. (2016). Recommendations for the evaluation of left ventricular diastolic function by echocardiography: An update from the American Society of Echocar-diography and the European Association of Cardiovascular Imaging. J. Am. Soc. Echocardiogr..

[6] Yamamoto J., Wakami K., Muto K., Kikuchi S., Goto T., Fukuta H., Seo Y., Ohte N. (2019). Verification of Echocardio-graphic Assessment of Left Ventricular Diastolic Dysfunction in Patients With Preserved Left Ventricular Ejection Fraction Using the American Society of Echocardiography and European Association of Cardiovascular Imaging 2016 Recommendations. Circ. Rep..

[7] Lancellotti P., Galderisi M., Edvardsen T., Donal E., Goliasch G., Cardim N., Magne J., Laginha S., Hagendorff A., Haland T.F. (2017). Echo-Doppler estimation of left ventricular filling pressure: Results of the multicentre EACVI Euro-Filling study. Eur. Heart J. Cardiovasc. Imaging.

[8] Inoue K., Khan F.H., Remme E.W., Ohte N., García-Izquierdo E., Chetrit M., Moñivas-Palomero V., Mingo-Santos S., Andersen Ø.S., Gude E. (2021). Determinants of left atrial reservoir and pump strain and use of atrial strain for evaluation of left ventricular filling pressure. Eur. Hear. J. Cardiovasc. Imaging.

[9] Obokata M., Kane G.C., Reddy Y.N., Olson T.P., Melenovsky V., Borlaug B.A. (2017). Role of diastolic stress testing in the evaluation for heart failure with preserved ejection fraction: A simultaneous invasive-echocardiographic study. Circulation.

[10] Solomon S.D., Claggett B., Lewis E.F., Desai A.S., Anand I.S., Sweitzer N.K., O’Meara E., Shah S., McKinlay S.M., Fleg J.L. (2016). Influence of ejection fraction on outcomes and efficacy of spironolactone in patients with heart failure with preserved ejection fraction. Eur. Heart J..

[11] Solomon S.D., McMurray J.J., Anand I.S., Ge J., Lam C.S., Maggioni A.P., Martinez F., Packer M., Pfeffer M.A., Pieske B. (2019). Angiotensin–Neprilysin Inhibition in Heart Failure with Preserved Ejection Fraction. N. Engl. J. Med..

[12] Yoshida T., Ohte N., Narita H., Sakata S., Wakami K., Asada K., Miyabe H., Saeki T., Kimura G. (2006). Lack of Inertia Force of Late Systolic Aortic Flow Is a Cause of Left Ventricular Isolated Diastolic Dysfunction in Patients With Coronary Artery Disease. J. Am. Coll. Cardiol..

[13] Kitada S., Kawada Y., Osaga S., Kato M., Kikuchi S., Wakami K., Seo Y., Ohte N. (2020). Left ventricular contractile performance and heart failure in patients with left ventricular ejection fraction more than 40%. Heart Vessel..

